# Controlling the Chemistry of Nanoclusters: From Atomic Precision to Controlled Assembly

**DOI:** 10.3390/nano12010062

**Published:** 2021-12-27

**Authors:** Srestha Basu, Anumita Paul, Rodolphe Antoine

**Affiliations:** 1 Schulich Faculty of Chemistry, Technion—Israel Institute of Technology, Haifa 3200003, Israel; srestha@iitg.ac.in; 2 Department of Chemistry, Indian Institute of Technology Guwahati, Guwahati 781039, Assam, India; 3 Institut Lumière Matière UMR 5306, Univ Lyon, Université Claude Bernard Lyon 1, CNRS, F-69100 Villeurbanne, France

**Keywords:** synthesis, purification, reactions, assembly, application, nanoclusters, ligands

## Abstract

Metal nanoclusters have gained prominence in nanomaterials sciences, owing to their atomic precision, structural regularity, and unique chemical composition. Additionally, the ligands stabilizing the clusters provide great opportunities for linking the clusters in higher order dimensions, eventually leading to the formation of a repertoire of nanoarchitectures. This makes the chemistry of atomic clusters worth exploring. In this mini review, we aim to focus on the chemistry of nanoclusters. Firstly, we summarize the important strategies developed so far for the synthesis of atomic clusters. For each synthetic strategy, we highlight the chemistry governing the formation of nanoclusters. Next, we discuss the key techniques in the purification and separation of nanoclusters, as the chemical purity of clusters is deemed important for their further chemical processing. Thereafter which we provide an account of the chemical reactions of nanoclusters. Then, we summarize the chemical routes to the spatial organization of atomic clusters, highlighting the importance of assembly formation from an application point of view. Finally, we raise some fundamentally important questions with regard to the chemistry of atomic clusters, which, if addressed, may broaden the scope of research pertaining to atomic clusters.

## 1. Introduction

Continuous efforts to achieve superior properties of assembled nanoscale particles have been limited due to the resulting poly-dispersity associated with colloidal routes of synthesis [1]. A solution to this limitation seems to have emerged from the advent of ligand-protected atomic clusters (often called nanoclusters or quantum clusters) [2,3]. In this case, ligands that stabilize the clusters are highly reactive in nature and thus provide facile avenue for “ligand mediated spatial organization of nanoclusters” [4,5,6]. Furthermore, the most important characteristics of atomic nanoclusters, which distinguish them from other classes of nanomaterials, is their structural integrity [7]. Notwithstanding the significant advancements achieved towards designing chemical routes to a synthesis of nanoclusters, the issue of poly-dispersity—*namely a collection of different ligand protected atomic cluster sizes*—remains a challenge to date. Thus, unlike other forms of nanoscale particles, a dispersion of atomic clusters typically constitutes of structurally and chemically related species. For example, a dispersion of nanoclusters may be purified following conventional separation techniques such as gel electrophoresis, size exclusion based ultrafiltration and normal and reverse phase chromatography [8]. Then, the precise chemical formula of the nanoclusters may be deciphered using mass spectrometry [9]. This has facilitated the use of nanoclusters in a plethora of applications such as bio diagnostics, therapeutics, catalysis and sensing, to mention a few of their uses [10,11,12,13,14,15,16].

Additionally, it has recently been demonstrated that the assembly of nanoclusters features superior physicochemical properties vis à vis their non-assembled analogues. This is primarily attributed to the collective properties of constituent nanoclusters in an “assembly” [17]. Thus, as evidenced by the recent upsurge of studies in the literature, [17,18,19] much attention is being paid towards the fabrication of complex nanostructures by using nanoclusters as building blocks. To this end, several strategies have been developed for the systematic organization of nanoclusters. For example, a straightforward method for assembling atomic clusters into assemblies has been pursued, based on the interaction among the ligands stabilizing the nanoclusters [20]. Similarly, the use of biological molecules such as DNA and proteins as templates for the organization of nanoclusters have also been reported. Notably, supramolecular interactions are also known to be instrumental in the formation of assembly of nanoclusters [21,22]. In this regard, the use of principles of coordination chemistry has been recognized as a possible route for the deterministic ordering of nanoclusters [21]. Formed assemblies of nanoclusters have been demonstrated to have enhanced stability, advanced property and superior application potential vis-a-vis the non-assembled clusters [23].

In this mini review, we aim to focus on the developments made so far with regard to the synthesis and applications of nanoclusters, their chemical reactions and eventual formation of their hierarchical assemblies endowed with superior application potential. This is deemed important to pave the way for the deterministic fabrication of tailor-made assemblies with desired functionalities. The current account is envisioned to not only enrich the scientific community with fundamental insights but also facilitate the advent of advanced nanostructures with controllable chemistry and tuneable properties. First, we focus on the plethora of strategies developed for synthesizing atomically precise clusters comprised of various metal atoms, stabilized by a definite number of ligands. To this end, we provide insight to the mechanism of formation of nanoclusters and thereby highlight the critical roles played by ligands in cluster synthesis. The purification, separation and isolation of nanoclusters is a necessary step between isolated clusters towards controlled self-assembly. We thus emphasise the importance of standard techniques that allow for the thorough purification of such clusters. Next, we discuss the rational strategies developed so far to add functionality to these nanoclusters, based on the principles of coordination and supramolecular chemistry [24]. The added functionality allows the nanoclusters to arrange into higher dimensional structures with superior physicochemical properties vis à vis in comparison to the non-assembled clusters. Thereafter, we justify the importance of assembly formation.

## 2. Ligand-Protected Metal Nanoclusters. Comparing Gold, Silver, Copper and Nickel

**Figure 1:**
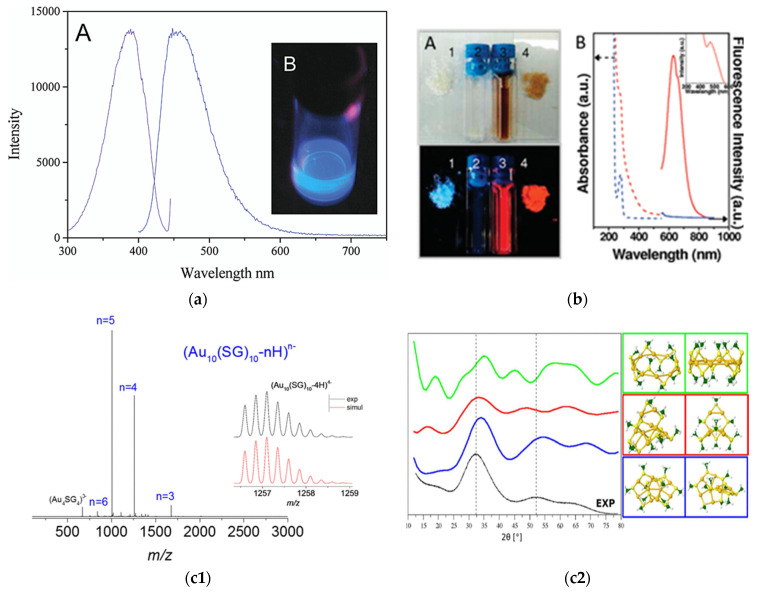
(**a**)**:** Excitation and emission spectra of Au_8_ nanodots. Inset shows digital photograph of Au_8_ nanodots, upon excitation at 366 nm. Reprinted with permission from [26]. Copyright 2003 American Chemical Society. (**b**) (A) Digital photographs of BSA stabilized Au NCs and control BSA (solution and powder) under visible and UV light. (B) Absorption and emission spectra of BSA stabilized Au NCs and control BSA. Reprinted with permission from [28]. Copyright 2009 American Chemical Society. (**c1**) Electrospray ionization (ESI) mass spectrum of Au_10_ clusters stabilized by glutathione. (**c2**) Experimental and simulated X-ray diffraction patterns of various isomers of Au_10_ clusters stabilized by glutathione. Reprinted with permission from [35]. Copyright 2017 American Chemical Society.

There has been a significant surge in efforts to synthesize atomically precise nanoclusters. The most explored metal nanocluster to date is that of gold. Gold is considered the noblest metal, and gold clusters are usually more stable than other noble metal clusters [25]. Gold nanoclusters have been synthesized using a multitude of stabilizers, including dendrimers, proteins, DNA, peptides and polymers. In this regard, Dickson et al. synthesized dendrimer-protected Au nanoclusters exhibiting a quantum yield as high as 40% [26]. Polyamido amine (4th generation) were used for the reduction and stabilization of Au_8_ nanoclusters (Figure 1a) [26]. On the other hand, natural bio macromolecules such as protein-stabilized gold nanoclusters have been found to be biocompatible [27]. Proteins such as Bovine serum albumin (BSA) and Human serum albumin (HSA) have been widely used for the stabilization of a gold nanocluster, comprising 25 Au atoms featuring red luminescence (Figure 1b) [28,29]. Other than these proteins, lysozymes, horseradish peroxidase and insulin have also been reported to stabilize gold nanoclusters [30,31,32]. DNA-templated gold nanoclusters have also been reported to feature bright luminescence, photo-stability and biocompatibility [33,34]. In addition, small molecules such as tripeptides (glutathione) (Figure 1c), [35,36,37] mercaptopropionic acid (MPA), [38] mercaptobenzoic acid (MBA), [39] penicillamine, [40] amino acids [41,42], etc., have been used to produce atomically precise gold nanoclusters. Interestingly, microorganisms such as bacteria have also been used as templates for the synthesis of Au NCs [43]. The key to the synthesis of gold nanoclusters is the exploitation of soft–soft interactions between gold and thiolated ligands, although nitrogenous ligands are also reported to be active in the stabilization of Au NCs in some cases. 

The synthesis of silver nanoclusters has always been considered as more challenging than the synthesis of their gold analogues. This is due to the greater tendency of zero-valent silver to become oxidized to their +1 oxidation state. Thus, much effort has been invested in the synthesis of a well-defined Ag nanocluster and in exploration of their properties at a sub-nanometer scale. Of all the strategies involved in the synthesis of Ag nanoclusters, the case of chemical etching of Ag nanoparticles has been recognized as the most popular one. This is largely due to the possibility of controlled Ag nanoclusters in mild etching environment. In this regard there are several reports where Ag NPs protected by mercapto-succininc acid (MSA) were etched to produce Ag_8_ and Ag_7_ NCs, which could further be separated using gel electrophoresis [44]. Additionally, red luminescent Ag NCs, comprising 38 Ag atoms, could be produced by etching off Ag NPs capped by citrate ions [45]. Another effective strategy employed for the synthesis of Ag NCs is based on ligand exchange. To this end, glutathione (SG) molecules stabilizing Ag_35_ NCs were exchanged with 4 fluorothiophenol to produce Ag_44_ NCs, stabilized by the latter ligand [46]. Notwithstanding the advantages of the above mentioned synthetic strategies the most effective method to obtain Ag NCs is through direct reduction of silver salts. 

**Figure 2:**
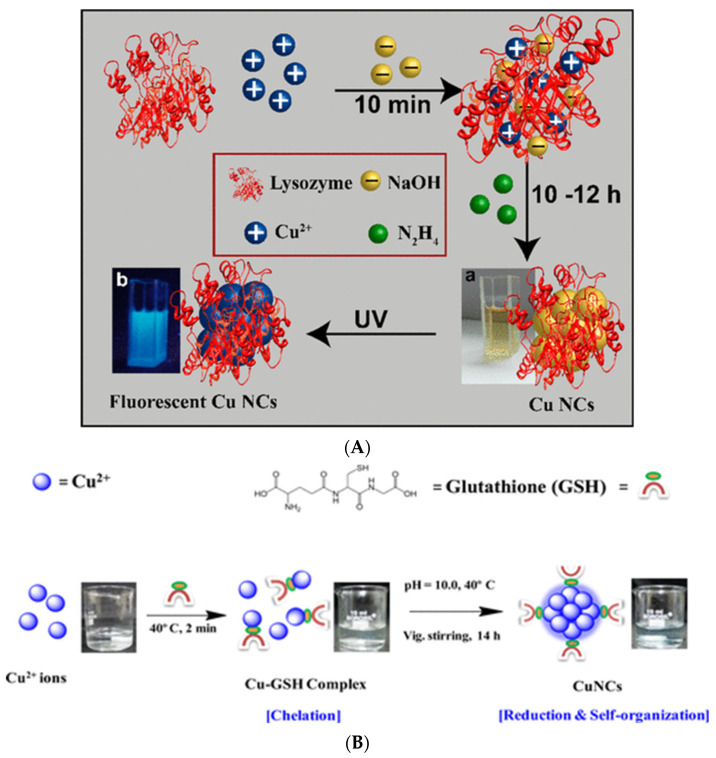
(**A**)**:** Schematic illustration for formation of Cu NCs stabilized by lysozyme. Reprinted with permission from [48]. Copyright 2014 American Chemical Society. (**B**) Schematic illustration for formation of Cu NCs stabilized by glutathione. Reprinted with permission from [55]. Copyright 2015 American Chemical Society.

Out of all classes of atomic clusters, copper-based nanoclusters were once considered to be extremely challenging. This is due to the reactive nature of copper, leading to the easy transformation of Cu (0) to Cu (II) in the presence of aerial oxygen. In this regard, in 2011, Chen and coworkers successfully reported the synthesis of copper nanoclusters comprising 8 Cu atoms stabilized by four thiolated ligands [47]. The route to the synthesis of the aforementioned clusters was based on mixing of Cu(NO_3_)_2_ and [N(oct)_4_][Br] in ethanol, following further treatment with 2-mercato-5N propyl pyrimidyl and reduction with NaBH_4_. This yielded atomically precise Cu clusters with near homogeneity in composition. On the other hand, highly luminescent Cu nanoclusters were synthesized using a ‘green’ approach, with protein as template. Specifically, copper precursors were incubated and reduced in the presence of proteins such as lysozymes, which eventually led to the formation of highly fluorescent Cu nanoclusters with a composition ranging from Cu_2_ to Cu_9_ (Figure 2A) [48]. Cu nanoclusters embedded in BSA [49] and HAS [50] have also been synthesized in an allied fashion. Additionally, other proteins, such as papain, [51] transferrin [52] and trypsin [53] have been used for the synthesis of Cu nanoclusters. A common feature that is typical with regard to the use of proteins in stabilizing Cu nanoclusters is that the synthetic procedure is associated with mixing the metal precursor with protein at ambient temperatures and with a basic pH. The use of an additional reducing agent such as ascorbic acid, NaBH_4_ is reported to have varied from procedure to procedure. DNA has been used to stabilize Cu nanoclusters featuring bright luminescence [54]. Moreover, small peptides such as L-glutathione, have been used to stabilize ultra-small Cu nanoclusters exhibiting a quantum yield as high as 8.6%. However, by using the same stabilizer, but varying the ratio of metal precursor to stabilizer Cu nanoclusters exhibiting blue luminescence have also been synthesized [55]. Other typical thiolated molecules such as thiosalicylic acid, [56] cysteine, [57] dihydrolipoic acid [58] and mercaptobenzoic acid [59] have been employed to synthesize Cu nanoclusters. When using thiolated molecules for the synthesis of copper nanoclusters, the stabilizer forms a complex with Cu(I) and the latter is reduced to Cu(0), either with the aid of additional reducing agents or by the thiol groups of the stabilizers themselves (Figure 2B). Copper nanoclusters exhibiting temporal, chemical and photo stability have been found suitable for applications ranging from catalysis, fluorescence based sensing of metal ions, disease markers, contaminants, pH, temperature, as vehicles for drug delivery, markers for cell labelling and as electrocatalysts, to name a few [60].

With regard to synthesis of Ni nanoclusters, the typical approach was to use carbonyl ligands as stabilizers. For example, [Ni_12-x_(PMe)_x_(CO)_24-3x_]^2-^ were synthesized using carbonyl protection [61]. However, carbonyl stabilized Ni clusters are typically negatively charged and thus counter cations are essential for their neutralization. Additionally, post synthetic functionalization of the synthesized clusters is difficult due to the presence of strongly bound carbonyl ligands. Thus, the advent of protected thiolate was deemed important for widening the chemistry of Ni based nanoclusters. To this end, Xu et al. performed a chemical reaction between NiCl_2_.6H_2_O and PhCH_2_CH_2_SH in a mixture of THF and methanol followed by reduction with aqueous NaBH_4_ to obtain Ni_39_ and Ni_41_ clusters [62]. Similarly, Ni_4_(PET)_8_ and Ni_6_(PET)_12_ [PET = phenylethane thiol] clusters were synthesized based on the ligand exchange strategy between glutathione-nickel complex and PET. The formed clusters were found to be effective electrocatalysts for water oxidation [63]. A table summarizing the synthetic details and properties of various nanoclusters is provided below (Table 1).

## 3. Purification, Separation, and Isolation of Nanoclusters

As synthesized NCs are often associated with the presence of NCs with varying compositions, namely, a dispersion of atomic clusters typically constitutes of structurally and chemically related species. This is, however, not desirable, as the properties of NCs are known to vary as a function of the number of atoms constituting the clusters, the structure of the overall clusters and the ligands (number density per cluster and nature) stabilizing the clusters. For example, clusters composed of fewer number of atoms are known to feature luminescence at lower wavelengths as opposed to clusters comprising of greater number of atoms. Similarly, physical properties of clusters such as solubility is dictated by the polarity of the ligands. Additionally, the catalytic activity of nanoclusters is highly dependent on the number density of the ligands stabilizing the clusters. Additionally, the application potential of nanoclusters becomes attenuated owing to the presence of unbound ligands in the reaction mixture. Thus, identifying and deciphering the chemical composition of a particular cluster (amongst a mixture of clusters produced synthetically) is of key importance to understand and modulate their application potential. In order to address this issue, several techniques have emerged for the purification of synthesized nanoclusters. Amongst the several purification techniques employed for the isolation of metal nanoclusters, the most common is the metal ion induced precipitation of nanoclusters. In this regard, Guan et al. successfully isolated BSA-stabilized Au_25_ NCs from unreacted BSA through Zn ion assisted precipitation of the former. In an allied vein, the pH of the reaction mixture containing the NCs can be adjusted to cause the precipitation of NCs. This method has also been used for the purification of BSA-stabilized Au NCs [64]. Precisely, the pH of the solution containing BSA stabilized Au NCs was reduced to reach the isoelectric point of BSA wherein the conformation of BSA was altered, leading to the agglomeration and eventual precipitation of BSA Au NCs. Similarly, methionine-protected Au NCs were separated following a reduction of the pH, leading to the relaxation of charge repulsion of the ligands, consequential aggregation of isolation of the NCs. Furthermore, the tuning of the solvent polarity of the reaction mixture containing the NCs has also been used for purification of NCs. For example, Galchenko et al. could selectively precipitate Au_25_ NCs by varying the ratio of methanol to water [65]. Another well-known strategy for the removal of unbound ligands is through the addition of ionic salts. This is because in the presence of additional ions, the zeta potential of the ligands stabilizing the NCs becomes reduced, which further led to the neutralization of charge and consequential aggregation of NCs. For example, mercaptoundecanoic acid (MUA) protected Au NCs are reported to aggregate and separate in presence of NaCl [66]. Thus, the use of external agents for the separation of NCs from unbound ligands, has emerged as a convenient customizable, rapid and cost effective technique for purification of NCs.

Apart from the aforementioned techniques, the isolation of NCs based on ‘size’ has also been widely practiced. For example, glutathione-protected Au NCs were separated from unreacted precursors using dialysis membranes with a cutoff of 3.5 k molecular weight [67]. The principal behind this technique is that glutathione molecules having smaller size vis à vis glutathione stabilized Au NCs could feasibly pass through the dialysis membrane, leaving the latter behind. Similarly, lipoic acid stabilized gold nanoclusters were purified from unreacted lipoic using a membrane of 3 kDa cutoff [68]. Furthermore, 14 kDa cutoff membranes were used to remove unreacted reactants from polyvinylpyrrolidone stabilized Cu NCs [69].

In order to isolate NCs of a particular composition from analogous NCs of varying composition, chromatographic methods have been sought after. The principle behind the technique is that clusters with polarity react with the stationary phase to a greater extent as compared to the less polar NCs. This would lead to faster elution of the non-polar NCs and greater retention of the polar NCs in the stationary phase. Using this principle Au_25_ NCs stabilized by phenyl ethane thiol were separated from Au_25_ stabilized by butane thiolate owing to difference of polarity of the respective ligands [70]. Glutathione stabilized clusters of varying composition such as Au_10_(SG)_10_, Au_15_(SG)_13_, Au_18_(SG)_14_, Au_22_(SG)_16_, Au_25_(SG)_18_, Au_29_(SG)_20_, Au_33_(SG)_22_, Au_39_(SG)_24_ were separated using the principle of reversed phase chromatography [71].

**Figure 3:**
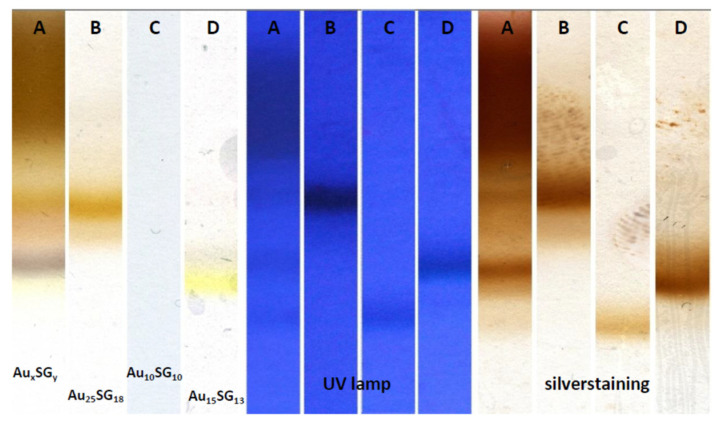
Polyacrylamide gel electrophoretic (PAGE) based separation of Au_10_(SG)_10_ from Au_15_(SG)_13_ and Au_25_(SG)_18_. Reprinted from Supporting Information Ref. [35]. Copyright 2017 American Chemical Society.

Electrophoretic techniques that involve separation, based on the differential mobility of charged species under the influence of an external electric field, have also been utilized for the separation of NCs of varying sizes. To this end, NCs with size difference of only 0.5 nm could be separated using gel electrophoresis. Importantly, the Antoine group recently demonstrated the use of polyacrylamide gel electrophoretic (PAGE) method to isolate Au_10_(SG)_10_ from Au_15_(SG)_13_ and Au_25_(SG)_18_ (see Figure 3) [35].

The above-described techniques are used for separating, purifying, and isolating a dispersion of atomic clusters, typically constituting different chemically related species. However, dispersions of atomic clusters for a given chemical composition can also comprise different but structurally related species. In such cases, molecular species in terms of 3-dimensional structures and structural dispersity, can be characterized, separated and even isolated based on the coupling of mass spectrometry with ion mobility spectrometry as recently demonstrated by Antoine group and other groups [9,72,73,74,75,76,77,78]. Thus, from the above discussion its it is apparent that synthetic and purification techniques of NCs have made great advancements in the last decade. This not only highlights the importance of research pertaining to atomic clusters but also presents them as ideal candidates for further chemical reactions and the advent of newer nanomaterials.

## 4. Chemical Reactions of Nanoclusters

### 4.1. Intermolecular Chemical Reactions of Nanoclusters. Example of Zinc Ion Induced Aggregation Strategy

Atomically precise nanoclusters are the closest analogues to molecules in classical chemistry. This is because, akin to molecules, atomic clusters have structural integrity, defined chemical composition and purity. Thus, in the same way that molecules are building blocks of compounds and bulk materials, nanoclusters are ideally suited as building blocks of hierarchical nanomaterials. This can be achieved through ‘chemical reactions’ of nanoclusters. However, unlike atoms and molecules which provide directed bonds, through covalent bonds for instance, nanoclusters are surrounded by an “isotropic” ligand shell devoid of such directional bonding. Therefore, in order to equip nanoclusters with directional bonding, it is essential that the nanoclusters are stabilized with chemically interacting ligands. This lays the foundation of chemical reactions of nanoclusters, either among themselves or with external chemical agents.

**Figure 4:**
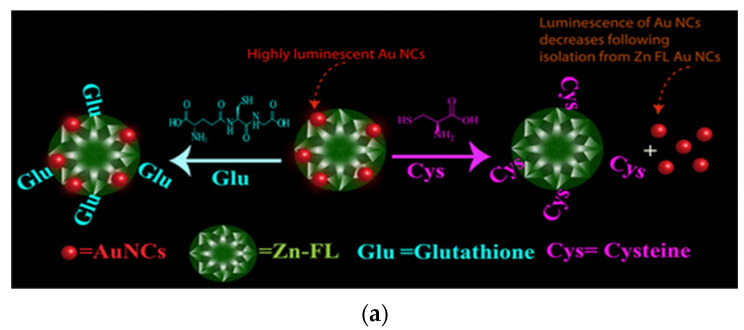

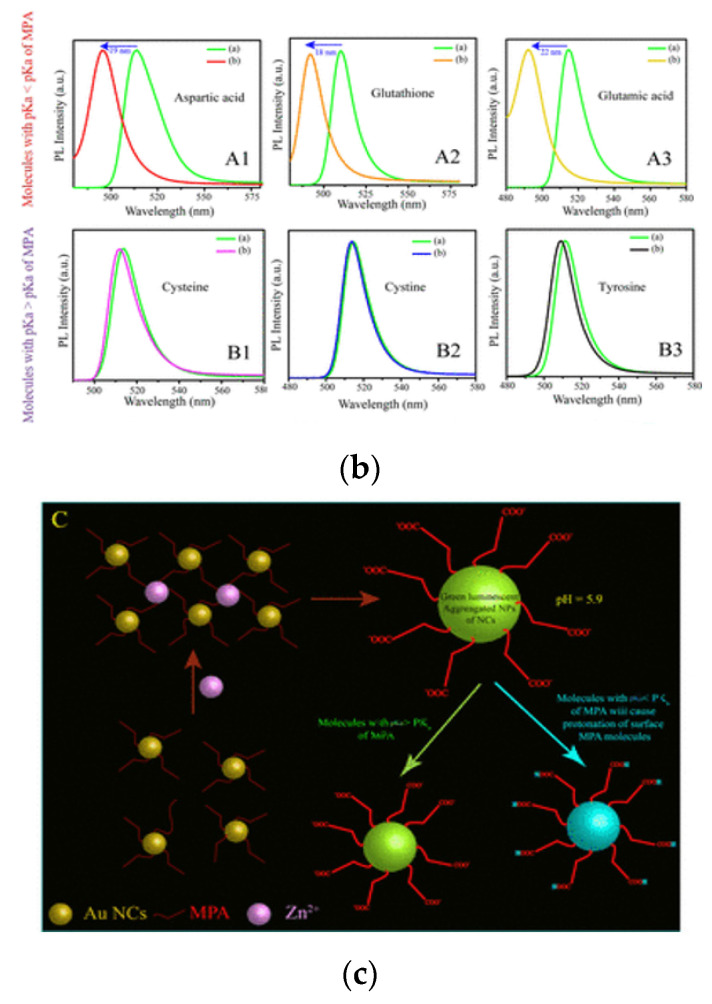
(**a**): Schematic illustration depicting possible mode of reaction among Au NCs-fluorescein composite and cysteine/glutathione. Reprinted with permission from [79]. Copyright 2018 American Chemical Society. (**b**) Variation in emission spectra of Zn Au NCs upon interaction with aspartic acid, glutathione, glutamic acid, cysteine, cystine and tyrosine. (**c**) Schematic illustration depicting possible mode of reaction among Zn Au NCs and aforementioned molecules. Reprinted with permission from [81]. Copyright 2019 American Chemical Society.

To this end, the Paul group has demonstrated a series of chemical reactions involving gold NCs. As a novel study, gold NCs were stabilized with MUA and were found to feature red luminescence. The Au NCs were then reacted among themselves via (bi)coordination between zinc ions and carboxylate terminals of the MUA molecules stabilizing the clusters. This led to an enhancement in the luminescence of Au NCs. Furthermore, (bi)coordinated zinc ions were coordinatively saturated following reactions with fluorescein molecules. The overall composite, constituting fluorescein and MUA stabilized Au NCs bridged with zinc ions, was rendered with dual red and green fluorescence. The chemical reactivity of the composite was then used to discriminate the biothiols, to the level of a few particles (Figure 4a) [79].

In another report, the chemical reactivity of histidine-stabilized Au NCs was exploited to perform intracellular logic operations. Briefly, the imidazole nitrogen of histidine moieties stabilizing Au NCs were conjugated with zinc ions, wherein the variation of luminescence of the former was suited to form a “tri state” logic operation at the cellular level. Furthermore, the zinc ion-coordinated histidine-stabilized Au NCs, when reacted with sulfide ions, were observed to quench the luminescence of the Au NCs, which could be used to construct an “on-off” intracellular switch. Finally, collective reactions among zinc ions, histidine stabilized Au NCs and sulfide ions, were used to form the basis of the “INHIBIT” gate within HeLa cells [80].

Additionally, zinc ions induced the aggregation of Au NCs, featuring bright green luminescence under visible light excitation, which were found to react differentially with amino acids, based on the pKa of the latter. This formed the basis for the discrimination of amino acids under visible light excitation using zinc ion-induced aggregates of Au NCs (Zn Au NCs) as a probe. The carboxylate ends of mercaptopropionic acid (MPA) stabilizing the clusters were coordinated with zinc ions, leading to the formation of green luminescent aggregates of Au NCs. The surface of the formed aggregates was further covered with MPA molecules. The pKa of MPA is reported to be 4.34. Thus, upon their reaction with amino acids with a pKa less than that of MPA, the surface MPA molecules became protonated, thereby blocking ligand to metal charge transfer, and quenching of the luminescence of Zn Au NCs. On the other hand, amino acids with a pKa greater than that of MPA did not react with the surface MPA molecules, thereby causing no discernible effect on the luminescence of Zn Au NCs. Thus, based on the differential reactivity of the MPA molecules present on the surface of Zn Au NCs towards amino acids with varying pKa, the latter could be distinguished easily (Figure 4b) [81].

To a similar end, the reactivity of the MPA molecules residing on the surface of allied Zn Au NCs probes, were also used to discriminate between geometrical isomers. However, unlike the case of amino acids, the proton-transfer reaction in the current report was further governed by intra molecular hydrogen bonding interactions among the analytes (Figure 4c) [81]. Interestingly, in a different study, Antoine and coworkers, measured the two photon excited fluorescence cross sections of the Zn Au NCs aggregates. Importantly, a four-fold enhancement in two-photon excited fluorescence of Zn Au NCs was observed vis à vis non assembled clusters. In the same study, for the first time ever, the molar mass of individual Au NCs recorded by time-of-flight mass spectrometry indicated the presence of Au_10_MPA_10_ catenane nanoclusters, while the entire mass distribution of aggregates of Au NCs was measured using charge-detection mass spectrometry [82].

### 4.2. Intramolecular Chemical Reactions of Nanoclusters. Example of Oxygen and Ligand and Metal Exchange Reactions

To focus on a different aspect, the luminescence of copper nanoclusters was modulated based on a reaction between the ligands (stabilizing the clusters) and aerial oxygen in presence of light. Briefly, Cu NCs stabilized with cysteine molecules, were found to feature red luminescence. The clusters, however, upon exposure to aerial oxygen in presence of light, were observed to become non luminescent. This was attributed to the desorption of cysteine molecules from the surface of Cu NCs, leading to the transformation of the latter into non luminescent aggregates. The cysteine molecules, in turn, were proposed to have transformed to S-nitrosothiolates following reaction with reactive nitrogen species [83].

**Figure 5:**
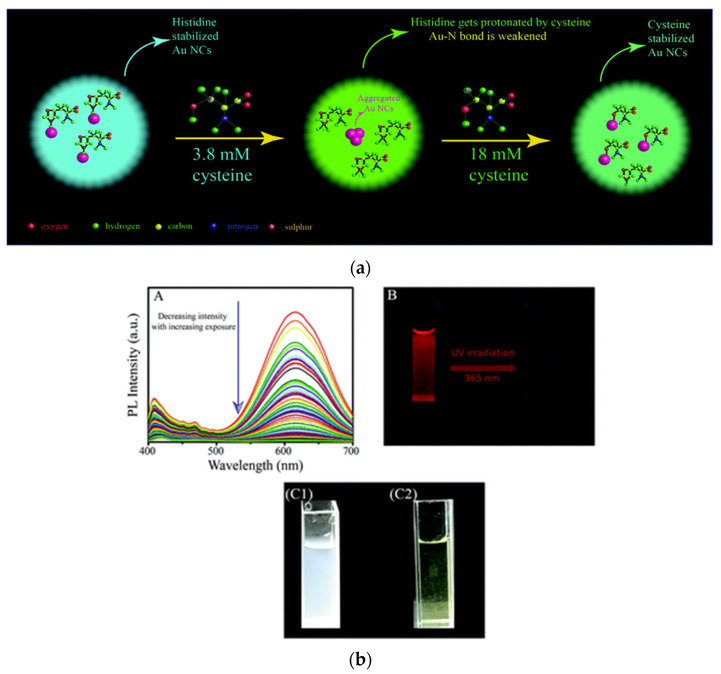
(**a**) Schematic illustration depicting ligand exchange reaction between histidine stabilized Au NCs and cysteine. Reprinted with permission from [84]. Copyright Royal Society of Chemistry 2020 (**b**) Experimental results showing quenching in luminescence of cystine stabilized Cu NCs upon reaction with oxygen in presence of light. Reprinted with permission from [83]. Copyright Royal Society of Chemistry 2019.

Additionally, ligand exchange reaction was performed between histidine stabilized gold nanoclusters and cysteine. The idea behind the study was that cysteine having thiol groups would replace the histidine molecules stabilizing the Au NCs, owing to greater aurophilicity of thiol groups in cysteine as compared to nitrogenous groups present in histidine moieties. This eventually led to alteration in luminescence of the clusters, as ligands stabilizing the clusters are well known for playing key role in the luminescence properties of the clusters. The ligand exchange reaction, involving Au NCs was well validated using X-ray photoelectron spectroscopy (XPS) (Figure 5a) [84].

For the first time, Antoine and co-workers performed a ligand exchange reaction of glutathione stabilized Au NCs-Au_15_(SG)_13_ with a single aminooxy ligand to yield Au_15_(SG)_12_(aminooxy)_1_. A detained mass spectrometric analysis revealed the successful ligand exchange of Au_15_(SG)_13_ by one aminooxy ligand. The ligand exchanged product served as a non-linear optical probe for the detection of protein carbonylation. This study was the first on the ligand exchange reaction for non-linear optical measurement based detection and quantification of protein carbonylation [85].

Furthermore, ligand exchange reactions have been reported to endow nanoclusters with achiral NCs with unprecedented chirality following exchange with chiral ligands. Burgi Yoshiki Niihori et al. successfully demonstrated that ligand exchange reactions between clusters and monothiolates commence at the terminal thiol groups of the thiolate protected NCs proceeding via an associative SN^2^ type mechanism [86].

Apart than reactions involving ligands present on surface of clusters, metal atom exchange leading to inter cluster reactions have also been pursued. In this regard, Pradeep et al. have demonstrated inter cluster reactions between Au_25_(SR)_18_ and Ag_44_(SR)_30_ where RS = alkyl/aryl thiolate [87]. Detailed experimental and theoretical analyses were performed to prove the occurrence of the metal exchange reaction. Additionally, this has been claimed to be the first ever report on inter cluster alloying. On a similar note, bimetallic NCs constituting Ag and Ni/Pd/Pt protected by dithiols were reacted with monothiol protected Au NCs [88]. This led to formation of tri-metallic NCs due to inter-cluster reactions between the starting clusters. In another report, Au_25_ NCs protected by PhCH_2_CH_2_S were reacted with Ag_25_ protected by 2,4-dimethyl benzene thiol to inter-cluster alloy. A detailed mechanistic investigation revealed that the reaction proceeded through two step metal exchange processes. In the first step, motif–motif exchange reaction leading to formation of ligand shell doped alloys of clusters occurred whereas in second step motif-motif and motif-kernel exchange reactions between the alloyed clusters took place. To this end, Antoine and co-workers reported the systematic doping of Au_10_ NCs stabilized by glutathione molecules. 1–3 silver atoms have been doped into Au_10_ NCs, displacing equivalent number of gold atoms from the clusters, which led to gradual variation in the two-photon excited fluorescence of the clusters [89].

The aforementioned examples of chemical reactions involving metal NCs clearly highlight their resemblance to the chemical reactivity of molecules. The knowledge gained from all these studies can be further extended to design new materials with tailored properties suited for diverse applications. This may start with the spatial organization of atomic NCs in higher dimensions. For example, ions, molecules and allied chemical species organized into three dimensions, i.e., their crystalline forms, are imbued with properties that are starkly different from that of the individual molecules. This is why crystalline forms of molecules are highly desired not only to gain fundamental insight into their chemistry but also for practical applications. Similarly, the constitutional precision of NCs and the ease with which they undergo chemical reactions provide great opportunities to expand the understanding and utility of NCs following assemblage into higher order dimensions.

## 5. Routes to Self-Assembled Structures of Nanoclusters. from Crystalline Assembly to Directed Assembly of NCs

Atomically precise clusters feature excellent biocompatibility, photo and temporal stability, catalytic activity, chemical reactivity, and tunable functionalities, and are thus envisioned to be model units for the fabrication of devices with an application potential ranging from catalysis optoelectronics energy storage and theranostics. However, pristine nanoclusters suffer from inherent limitations such as low quantum yield (in general), low stability under harsh conditions such as extremes of pH, heat and solvent. Often, clusters tend to aggregate into undefined moieties thereby losing their integral physicochemical characteristics. This restricts their application potentials. A possible solution for this issue is likely to emerge from the systematic organization of these nanoclusters into deterministic structures. This could be advantageous in several fronts, including gaining complete insight into the structures of NCs, delineating their structure property relationship, fine tuning their structures to tailor their properties for a desired application, conferring them with added stability and making them versatile for practical utilities. In this regard, several efforts have been directed to spatially organize NCs into well-defined structures.

As stated in earlier sections, the ligands stabilizing the clusters play a determining role in not only controlling their interactions but also additional chemical agents. For instance, the charges on the ligands keeps the individual cluster units apart from each other and prevent their uncontrolled agglomeration. Likewise, ligands with functional groups capable of hydrogen bonding interact among themselves to confer stability to clusters. On the other hand, ligands with terminal groups capable of chemical coordinating with metal ions, provide a facile platform for a complexation reaction mediated assembly of NCs.

**Figure 6:**
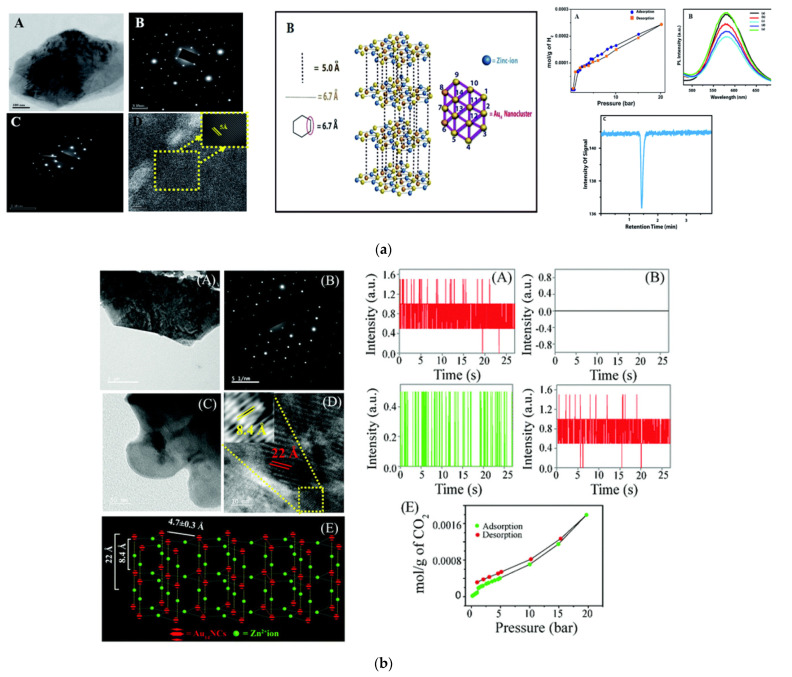
(**a**) Experimental results showing formation zinc mediated three-dimensional crystalline assembly of Au NCs stabilized by MPA and histidine, and use of the assembly in storage and sensing of hydrogen at plaint conditions. Reprinted with permission from [90]. Copyright Royal Society of Chemistry 2016. (**b**) Experimental results showing formation zinc mediated three-dimensional crystalline assembly of Au NCs stabilized by MPA and cysteine, and use of the assembly in storage and sensing of carbon dioxide at a few particle level. Reprinted from Ref. [94] with permission. Copyright Royal Society of Chemistry 2018.

In this regard, Chattopadhyay and coworkers and Paul and coworkers demonstrated, for the first time, that the complexation reaction between ligands stabilizing gold NCs and zinc ions can lead to the formation of three to two dimensional crystalline assemblies of clusters. Interestingly, they have shown MPA and Histidine stabilized Au_14_ NCs upon reaction with Zn ions results in the formation of three-dimensional assembly of the clusters. The three-dimensional structure derived from transmission electron microscopic (TEM) and selected area electron diffraction (SAED) analysis. The formed 3D assembly of Au NCs was further used for storage and sensing of hydrogen gas at ambient conditions of 20 °C and 20 bar (Figure 6a) [90]. In a similar fashion, two dimensional crystalline nanosheets were formed out of reaction between Au_14_ NCs (stabilized with L phenylaniline and MPA) and zinc ions. The two dimensional nanosheets were used for reversible storage of oxygen at pliant conditions of 20 °C and 20 bar pressures [91].

Similarly, MPA and L/D tryptophan stabilized Au NCs were assembled into three-dimensional organization following complexation reaction amongst Zn ions and carboxylate groups of MPA and L/D tryptophan. The resultant assembly was characterized using TEM, SAED and powder XRD, which revealed their crystalline nature. The crystalline assemblies of Au_14_ clusters were then used for fluorimetric chiral recognition and separation of externally added L and D tryptophan. Interestingly, it was observed that when Au_14_ clusters were stabilized with L tryptophan, the resultant crystalline assembly was responsive to L tryptophan only. Similarly, D tryptophan stabilized Au14 clusters constituting the crystalline assembly was responsive to D tryptophan only. The basis of chiral recognition and separation of tryptophan by the crystalline assembly was attributed to the classical three-point vs. two-point interactions between tryptophan analytes and the crystalline assembly. It is important to note here that, as opposed to the assembled clusters, non-assembled Au clusters were observed to be nonresponsive to the chiral behavior of L and D tryptophan [92].

MPA and L tyrosine-stabilized Au NCs were assembled into nanocrystals following coordination of the carboxylate groups of the ligand and zinc ions. The so formed nanocrystals were characterized using analytical techniques such as TEM and SAED. The crystalline assemblies were found to be effective for mitochondria-targeted cancer theranostics with the rare potential of facile renal clearance. The anticancer activity of the zinc mediated assembly was attributed to the generation of reactive oxygen species within HeLa cells [93].

Moreover, MPA and L cysteine stabilized Au_14_ NCs upon complexation reaction with zinc ions led to the formation of allied crystalline assembles, which could also be successfully characterized with TEM and SAED techniques. Importantly, the non-assembled Au_14_ clusters were found to feature photoluminescence intermittency when illuminated at few particle level, whereas the zinc ion-mediated assembly of clusters was found to be non-blinking under identical conditions. This was attributed to the fact that the non-radiative relaxation pathways active in non-assembled clusters, due to the conformational relaxation of the ligands, were blocked due the structural rigidity gained by the clusters upon complexation with zinc ions in the assembled structure. Additionally, in contrast to non-assembled clusters, the crystalline assembly of Au_14_ clusters could be used for storage of CO_2_ and sensing of the latter at a few particle level (Figure 6b) [94].

MPA and MBA stabilized Au_14_ nanoclusters assembled into two dimensional organizations, following the complexation reaction with zinc ions was demonstrated to exhibit delayed fluorescence with ultra-long luminescence lifetime of 0.5 ms and quantum yield of ~19%. This marked the advent of the first ever report of crystalline assembly of gold nanoclusters exhibiting lifetime in sub millisecond range. The occurrence of delayed fluorescence was proposed to have originated from the restricted intramolecular motion of the ligands stabilizing the clusters (in assembly), which consequently led to reduced non radiative transitions otherwise occurring in non-assembled Au_14_ clusters [95].

The aforementioned reports of complexation reaction mediated assemblage of Au_14_ nanoclusters with synergistic properties of nanoscale clusters as well as inorganic complexes portends to form a new class of hierarchical nanomaterials concerted with futuristic application potential. Additionally, as opposed to assemblies of clusters, devoid of periodicity, crystalline assemblies are envisioned to have uniform chemical properties and a predictable application potential.

In addition to the principles of coordination chemistry, other strategies have also been adopted for the construction of an assembly of nanoclusters. In this regard, assemblies related to supramolecular chemical interactions are worth mentioning. An important example is provided by Yoon et al., who performed a large scale preparation of super crystalline lattices of [Na_4_Ag_44_(p-MBA)_30_]. Hydrogen bonding amongst the carboxylate groups of the ligands was recognized as the key to form rhombus-shaped super crystals of [Na_4_Ag_44_(p-MBA)_30_] [96]. Single crystal X-ray crystallography revealed that the layers of the super lattice were connected through 24 and 36 intra and inter layer hydrogen bonds, respectively. Yao et al. constructed super structures of [Ag_44_(p-MBA)_30_]^4-^ NCs, following the interaction of the carboxylate ends of the ligands with appropriate counter ions [97]. Zhang et al. fabricated the self-assembly of glutathione stabilized Au NCs, which closely mimics the self-assembly of capsid proteins. In this study, the initial disruption of hydrogen bonding between glutathione and water molecules was achieved following introduction of DMSO. Eventually, hydrogen bonding among glutathione molecules stabilizing the clusters was promoted through the subsequent removal of water molecules, which led to the formation of large spherical assemblies of Au_22_ NCs [98].

In addition to hydrogen bonding, other interactions such as electrostatic interactions, have also been utilized for the fabrication of self-assembled structures of NCs. The key idea behind this approach is the proper balance of charges among the ligands present on the surface of neighboring clusters, such that the clusters should neither undergo uncontrolled agglomeration nor should they remain too apart to hinder self-assembly. In order to construct an assembly of clusters, electrostatic interactions are often used in conjunction with other driving forces such as hydrogen bonding and chemical coordination, to maintain the delicate balance of proximity of the constituent clusters. Moreover, van der Waals interactions based assemblies of NCs are also known. For instance, dodecane thiol stabilized Au_15_ clusters were self-assembled into distinct nanosheets at elevated temperatures [99]. Hydrophobic interactions among the non-polar chains of dodecane thiol were proposed to have played a key role in the assemblage process.

In addition to self-assembly, a directed assembly of NCs have also been pursued. In this regard, templates, either stabilizing the clusters or externally added to the medium, have been proposed to play the determinant role in the assemblage process. To this end, macromolecules such as DNA, [100] macrocycles, [101] synthetic and natural polymers, [102] functionalized graphene oxide [103] have been reported to be instrumental in guiding the assembly of NCs. The chief idea in this approach is to allow concomitant binding of the NCs to the templates as well as spatial organization of the former in a way directed by the latter. For example, polymers imbued with appropriate functionalities can be tailored to interact with NCs in a specific manner, followed by hierarchical organization of the clusters into a geometry guided by the polymer. In this regard, nanospheres exhibiting aggregation induced emission have been fabricated via polyethyleneimine guided self-assembly of Ag(I) NCs [104]. Further, multi-thiolated co-polymers were used as scaffold for synthesis of Cu NCs. This was claimed to not only render the as synthesized NCs resistant towards oxidation and uncontrolled aggregation, but also imbued the clusters with thermo and pH responsive properties of the pristine co-polymers. The so formed polymeric hybrid structure of NCs was further used for sensing of Hg ions [105]. Moreover, natural biopolymers such as bacterial proteins have been used to self-assemble glutathione stabilized NCs. These nanocomposites could further be used as agents for cell imaging and cargo for protein delivery [106]. However, assembly induced by polymers often lead to uncontrolled agglomeration of clusters which render them inappropriate for practical applications.

This issue is often circumvented by the use of DNA as a template for assemblage of NCs. This is primarily attributed to the fact that DNA templates provide the combined options of deterministic structural motif as well as intrinsic binding sites, namely, the nucleobases. As a consequence, several nano-architectures have been constituted following the DNA-guided assembly of atomic clusters. For example, DNA nanoribbons constituting appropriate binding sites have been used as a template for synthesis cum assembly of ultra-small Cu NCs. DNA assembled Cu NCs were reported to feature aggregation induced emission attributed to structural rigidity gained by Cu NCs upon being assembled by DNA [107]. Furthermore, Wang and coworkers assembled Au NCs in the presence of double stranded DNA and used them for cancer theranostics [108]. Similarly, Chattopadhyay and co-workers synthesized Au NCs using double stranded DNA as templates, mimicking the process of polymerase chain reaction. Further luminescence intensity of the clusters could be used to probe and quantify double stranded DNA, i.e., the PCR products [34].

Moreover, macrocyclic molecules have been appropriately tailored to interact with the ligands of the atomic clusters to produce rigid assemblies featuring alluring photo physical properties. For example, Au_22_ NCs stabilized by peptides of sequence Phe-Gly-Gly-Cys were reported to undergo non-covalent interactions with Cucurbiturils, leading to the formation of rigid assemblies exhibiting enhanced quantum yield as opposed to non-assembled clusters [101]. Moreover, Ag_29_ NCs stabilized by 1,3-benzene dithiol and triphenyl phosphine were reported to react with crown ethers, leading to crystallization induced organization of the crown ether molecules into the interstitial sites of the lattice of Ag_29_ NCs [109]. The crystalline co-assembly of Ag_29_ NCs were found to be 3.5 times more luminescent than for the non-assembled clusters. Likewise, an aggregation-induced emission probe, constituting cyclodextrin functionalized Cu NCs, and di(adamantan-1-yl) phosphine, was designed for in situ imaging of membrane associated glycoproteins [110].

Additionally, external stimuli such as pH have been used to trigger assemblies of ultra-small copper nanoclusters. For instance, Cu NCs stabilized by L cysteine were found to undergo reversible aggregation and disaggregation, as a function of pH. Monodispersed Cu NCs were found to form insoluble macroscopic aggregates featuring red luminescence at pH ~ 3, whereas the aggregates were reported to disintegrate into soluble dispersion, showing weak luminescence at pH > 4. Similarly, orange-red emitting Cu NCs were spontaneously self-assembled into nano-spheres, nano-meshes and nanosheets by fine tuning the extent of hydrogen bonding interactions among m-amino thiophenols stabilizing the Cu NCs clusters [111]. A further development was achieved by Chattopadhyay and co-workers, who reported the synthesis of pH responsive Cu NCs, featuring aggregation-induced emission within cancer cells. Cu NCs were reported to exhibit orange-red emission at pH 4.5, whereas the same Cu NCs, following intracellular aggregation, featured bright green luminescence at pH 7.4. Interestingly, the rate constants for intracellular aggregation of Cu NCs were found to be different for different cancer cell lines [112].

## 6. Concluding Remarks

Atomically precise nanoclusters, once considered to be “just another” luminescent nanomaterial, has now become the heart and soul of research pertaining to nanoscale science and technology. This, as discussed in the earlier sections, is primarily attributed to their chemical and structural integrity. Thus, nanoclusters are being widely explored as model analogues of molecules in classical chemistry. They are often called supertoms or superatomic molecules [113,114,115]. Significant resources are being invested in designing rational and facile strategies for the synthesis of atomic clusters. Purification techniques are coming up to isolate ultra-pure atomic clusters [8]. Chemical reactions are being performed on atomic clusters to form “products” akin to reactions of molecules and compounds. Research is being directed to elucidate the mechanism of such reactions, gaining insights into the intermediates formed and maximizing the yield of the products. Further, controlled assemblies of atomic clusters are being fabricated using various principles of chemistry ranging from coordination to supramolecular. The as-described assemblies of nanoclusters have found finding applications in wide areas ranging from catalysis to theranostics.

In this mini review, we aimed to summarize the recent advancements made with regard to all these aspects of atomic clusters. Additionally, in the conclusion, we raise questions, which, if addressed in the near future, may further illustrate the versatility of atomic clusters. Firstly, it is important to elucidate the relationship between the number of atoms constituting the clusters and their corresponding properties (optical and chemical). For instance, it is well known that clusters with less atoms feature luminescence at wavelengths lower than the emission wavelengths of clusters with comparatively more atoms. However, an exact relationship between the number of atoms composing the clusters and the resultant emission properties is not fully understood. This is largely because, in addition to the number of atoms constituting the clusters, the ligands stabilizing the clusters also play key roles in determining their luminescence properties [116]. Thus, the emergence of generalized principles governing the properties of clusters is deemed essential. Clearly, quantum chemistry methods elucidating the geometry of the clusters and configuring their optical properties in terms of molecular transitions should be conducted in further depth for the assembly of clusters, and innovative approaches such as the hybrid QM/MM (quantum mechanics/molecular mechanics) approach might address their structure–properties relationships [117,118,119,120,121].

Secondly, with regard to the reactions of atomic clusters, the study of chemical kinetics has remained largely unexplored. Additionally, in classical chemistry, the mystery of “bond breaking and making” in molecules has been, and continues to be, gradually unveiled. However, such mysteries continue to prevail with regard to the chemistry of atomic clusters. These studies, if performed, may open up newer domains to control the yield of reactions, optimize the conditions of reactions, trap the essential intermediates, and identify the transition states of relevant reactions.

Thirdly, with regard to the controlled assembly of clusters, delineation of a definite structure property relationship is deemed important. Notwithstanding the robustness of the strategies for systematic organization of clusters developed so far, little effort has been directed towards deciphering a “one to one” correlation between the structures of assembled clusters and their emerging properties. This is not only important to enhance the ease of assemblage of nanoscale clusters, but also to widen their application potential. Finally, in an assembly of clusters, it is important to understand whether the ultimate properties are an outcome of additive behavior of the individual clusters, or a result of a synergistic behavior between the clusters, ligands, and the templates.

## Figures and Tables

**Table 1:** Summary of the synthetic details and properties of various nanoclusters (presented in this review).

Metal	Stabilizer	No. of Metal Atoms Comprising the Clusters	Emission Color	Reference
gold	Polyamido amine (4th generation)	8	blue	26
gold	BSA	25	red	28
gold	HSA	25	red	29
gold	DNA	7	red	33
gold	glutathione	10	Non-emissive	35
gold	MPA + chitosan	20	orange-red (pH dependent)	38
gold	histidine	10	blue	41
gold	MPA + bacteria	Not determined	white	43
silver	produced by Ag NPs protected by mercapto-succininc acid	8, 7	red, blue green	44
silver	produced by etching of Ag NPs capped by citrate ions	38	red	45
copper	lysozymes	2–9	blue	48
copper	glutathione	15	blue	55
copper	cysteine	4	cyan	57
copper	dihydrolipoic acid	4	red	58
nickel	phenylethanethiol	4, 6	Not reported	63
